# Candidalysin Inhibits *Porphyromonas gingivalis* Lipoprotein-Induced IL-1β Production in BV-2 Microglia via Hydrophobic Microbial Interactions

**DOI:** 10.3390/ijms27156614

**Published:** 2026-07-24

**Authors:** Haruka Kanagawa, Ayaka Kawahara, Nene Mikawa, Kana Sugihara, Momoha Ueda, Ayano Nitta, Mizuki Egi, Reina Oda, Saori Nonaka, Hidetoshi Tozaki-Saitoh, Kosuke Oda, Hiroshi Nakanishi

**Affiliations:** 1 School of Pharmacy, Yasuda Women’s University, Hiroshima 731-0153, Japan23141218@st.yasuda-u.ac.jp (A.K.); 23141239@st.yasuda-u.ac.jp (N.M.); 23141227@st.yasuda-u.ac.jp (K.S.); 23141105@st.yasuda-u.ac.jp (M.U.); 2414128@st.yasuda-u.ac.jp (A.N.); 24141104@st.yasuda-u.ac.jp (M.E.); 23141209@st.yasuda-u.ac.jp (R.O.); 2 Laboratory of Molecular Pathobiology, Faculty of Pharmaceutical Sciences, Suzuka University of Medical Science, Mie 513-8670, Japan; nonaka-s@suzuka-u.ac.jp; 3 Department of Pharmaceutical Sciences, School of Pharmacy at Fukuoka, International University of Health and Welfare, Fukuoka 831-8501, Japan; saitoh@iuhw.ac.jp; 4 Department of Pharmacotherapeutics, Faculty of Pharmacy and Pharmaceutical Sciences, Fukuyama University, Hiroshima 729-0292, Japan; oda-k@fukuyama-u.ac.jp; 5 Department of Pharmacology, Faculty of Pharmacy, Yasuda Women’s University, Hiroshima 731-0153, Japan

**Keywords:** BV-2 microglia, candidalysin, hydrophobic interactions, interleukin-1β, lipopolysaccharide, nuclear factor-κB, outer membrane lipoproteins, outer membrane vesicles, *Prophyromonas gingivalis*

## Abstract

In postmortem Alzheimer’s disease (AD) brains, *Porphyromonas gingivalis* (*Pg*), a major periodontal pathogen, and *Candida albicans*, one of the most common fungal pathogens, have been detected. Although it is important to better understand the effects of their co-infection in the brain for elucidating the pathogenesis of AD, little is known about the neuropathological significance of such co-infection. In the present study, we aimed to elucidate the effects of co-exposure to virulence factors derived from *Pg* and *C. albicans* on microglial inflammatory responses. We demonstrated, for the first time, that both candidalysin dissolved in dimethyl sulfoxide (CLd) and water (CLw) significantly suppressed *Pg* lipopolysaccharide (LPS)-induced interleukin-1β (IL-1β) production by 35–60% and nuclear factor-κB (NF-κB) activation by 20–40%. It should be noted that contaminating *Pg* outer membrane lipoproteins in *Pg* LPS were mainly responsible for IL-1β production. To examine the possible hydrophobic interactions between lipoproteins contaminating the *Pg* LPS preparation and CL, we used 8-anilino-1-naphthalenesulfonic acid sodium salt (ANS-Na), which can be excited to emit fluorescence by binding of hydrophobic molecules. The mean fluorescence intensity of ANS-Na was significantly reduced by approximately 26% following co-treatment with CLw and *Pg* LPS compared with CLw alone. Furthermore, we generated a mutant form of CL with reduced hydrophobicity (GRAVY index: 1.106 vs. 0.874) while preserving its predicted structural properties. This mutant CLd no longer inhibited *Pg* LPS-induced IL-1β production. Taken together, these findings indicate that hydrophobic interactions between lipoproteins contaminating the *Pg* LPS preparation and CL mediate the inhibitory effect of CL on *Pg* LPS-induced inflammatory responses. The present findings suggest that interactions between polymicrobial virulence factors in the brain may modulate microglia-mediated inflammatory responses during AD progression.

## 1. Introduction

There is growing clinical evidence that chronic periodontitis is closely linked to the initiation and progression of Alzheimer’s disease (AD). Both subjects with AD and those with mild cognitive impairment exhibited significantly elevated serum IgG antibodies to *Prevotella intermedia* and *Fusobacterium nucleatum* at baseline, prior to the diagnosis of neurological changes [1]. Furthermore, subjects with AD exhibited significantly elevated serum IgG antibodies against *Treponema denticola* and *Porphyromonas gingivalis* (*Pg*) at baseline. The three major periodontal pathogens comprising the so-called “red complex”, consisting of *T. denticola*, *Tannerella forsythia* and *Pg*, and/or their bacterial components, have been detected in the brain tissues of individuals with and without dementia [2]. In addition to periodontopathic bacteria, other microorganisms, including *Candida albicans*, one of the most common fungal pathogens, have also been detected in postmortem AD brains [3,4].

It has been reported that *C. albicans* enhances the virulence and survival of *Pg* under an anaerobic microenvironment [5]. *C. albicans* also attracts immune cells, such as neutrophils and macrophages, which may facilitate the endocytosis of *Pg* [6]. Moreover, macrophages simultaneously exposed to *Pg* and *C. albicans* produce higher levels of pro-inflammatory cytokines [7]. Therefore, examining the coexistence of periodontopathic bacteria and other microorganisms is important to better understand the pathogenesis of AD. However, little is known about the pathological significance of co-infection of *Pg* and *C. albicans* in the brain.

*Pg* possesses several virulence factors, including lipopolysaccharide (LPS), outer membrane lipoproteins, cysteine proteases known as gingipains, and outer membrane vesicles (OMVs) as their carriers. Secreted gingipains facilitate the entry of *Pg* and its virulence factors into the brain by degrading tight junction proteins of the blood–brain barrier (BBB) [8]. Chronic systemic exposure to *Pg* LPS induces AD-like pathologies in mice, including microglia-mediated neuroinflammation and cognitive decline [9,10]. Furthermore, *Pg* LPS, lipoproteins and OMVs induce interleukin-1β (IL-1β) production in microglia through distinct mechanisms [11,12]. On the other hand, *C. albicans* secretes several virulence factors, including secreted aspartic proteases and candidalysin (CL), a hydrophobic peptide toxin. Secreted aspartic proteases mediate cerebral invasion of *C. albicans*, at least in part, through degrading tight junction proteins in BBB endothelial cells [13]. Mice infected with *C. albicans* exhibit mild memory impairment, microglial activation, and increased production of pro-inflammatory cytokines [14]. Moreover, CL can induce inflammatory responses through activation of microglia and macrophages [13,15,16,17,18].

In the present study, we have thus attempted to elucidate the effects of co-exposure to virulence factors derived from *Pg* and *C. albicans* on inflammatory responses in microglia. Here, we demonstrate for the first time that CL significantly suppressed IL-1β production and nuclear factor-κB (NF-κB) activation induced by outer membrane lipoproteins contaminating the *Pg* LPS preparation through hydrophobic interactions. The present findings reveal a previously unrecognized mechanism of neuroinflammatory regulation mediated by polymicrobial interactions, in which CL, despite being a cytolytic peptide toxin, exerts anti-inflammatory effects.

## 2. Results

### 2.1. Effects of CL on the Production of IL-1β Induced by Pg LPS in BV-2 Microglia

CL contains hydrophobic residues such as Ile and Leu at its *N*-terminal region and hydrophilic residues such as Lys and Asn at its *C*-terminal region, allowing it to dissolve in both organic solvents and water. Therefore, two forms of CL were used: CLd, dissolved in dimethyl sulfoxide (DMSO), and CLw, dissolved in purified water.

**Figure 1:**
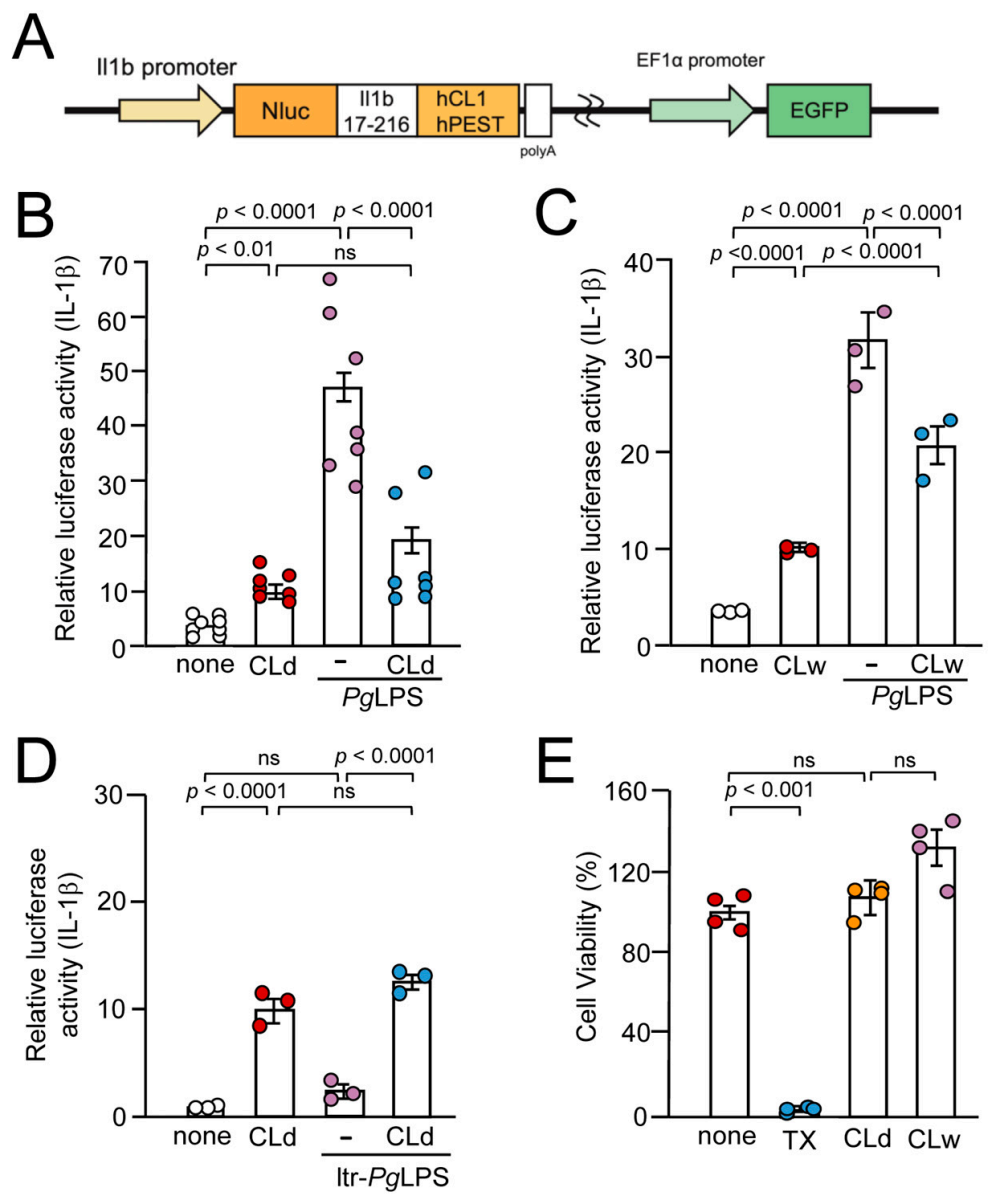
A schematic illustration of the IL-1β probe construct and effects of CL on *Pg* LPS-induced IL-1β production in BV-2 microglia at 1 h after stimulation. (**A**) The *Nluc* luciferase gene was fused with the sequence corresponding to the IL-1β cleavage site (*Il1b* 17-216). The *C*-terminus of the IL-1β cleavage site was further fused to two protein destabilization sequences (hCL1 and hPEST). The resulting fusion gene was ligated downstream of the mouse IL-1β promoter. (**B**) Mean relative luciferase activity of the IL-1β probe following treatment with CLd (10 μM), *Pg* LPS (30 μg/mL), or their co-application. (**C**) Mean relative luciferase activity of the IL-1β probe following treatment with CLw (100 μM), *Pg* LPS (30 μg/mL), or their co-application. (**D**) Mean relative luciferase activity of the IL-1β probe following treatment with CLd (10 μM), lipase-treated *Pg* LPS (ltr-*Pg* LPS, 30 μg/mL), or their co-application. (**E**) Mean cell viability (%) of BV-2 microglia after 1 h exposure to CLd (10 μM) or CLw (100 μM). Triton X-100 (TX, 0.1%) was used as a positive control. “-” indicates vehicle treatment (no CL exposure, DMSO alone for CLd experiments and water for CLw experiments). Data are presented as individual data points from 3 to 7 independent experiments with the mean ± SE. Each dot represents one independent experiment. Statistical analyses were performed using one-way analysis of variance followed by Tukey’s post hoc test. ns, not statistically significant.

IL-1β levels were quantified by measuring luciferase activity in IL-1βNanoLuc (Nluc) reporter BV-2 microglia (Figure 1A). *Pg* LPS-induced luciferase activity peaked at 1 h after stimulation and returned to basal levels within 6 h [12]. Therefore, the effects of CLd on *Pg* LPS-induced production of IL-1β were examined at 1 h after stimulation. *Pg* LPS (30 μg/mL) significantly increased luciferase activity in IL-1βNluc reporter BV-2 microglia at 1 h after stimulation. In contrast, the effect of CLd (10 μM) alone on IL-1β production varied across experiments. In some experiments, CLd modestly but significantly increased luciferase activity (Figure 1B,D), whereas in others, it did not induce a statistically significant increase in IL-1β production. Notably, co-treatment with CLd and *Pg* LPS consistently and significantly reduced IL-1β production compared with *Pg* LPS treatment alone (Figure 1B).

Previous studies reported that the bioactivity of CLw is approximately 10-fold lower than that of CLd, probably because CLw mostly aggregated to form microparticles and was only partly soluble to form nanoparticles [19]. Therefore, CLw was used at 10-fold higher concentration than CLd. Similar results were obtained with CLw (100 μM). CLw modestly but significantly increased luciferase activity in IL-1βNluc reporter BV-2 microglia (Figure 1C), although this increase did not reach significance in some experiments. As observed with CLd, co-treatment with CLw and *Pg* LPS consistently and significantly reduced IL-1β production compared with *Pg* LPS treatment alone (Figure 1C).

Lipase-treated *Pg* LPS induced only low levels of IL-1β production in BV-2 microglia (Figure 1D), suggesting that *Pg* outer membrane lipoproteins contaminating the *Pg* LPS preparation are primarily responsible for IL-1β production in BV-2 microglia. CLd showed no significant effect on IL-1β production induced by lipase-treated *Pg* LPS (Figure 1D). Furthermore, neither CLd (10 μM) nor CLw (100 μM) showed significant cytotoxic effects on BV-2 microglia at 1 h after treatment (Figure 1E).

### 2.2. Effects of CL on the Activation of NF-κB Induced by Pg LPS in BV-2 Microglia

**Figure 2:**
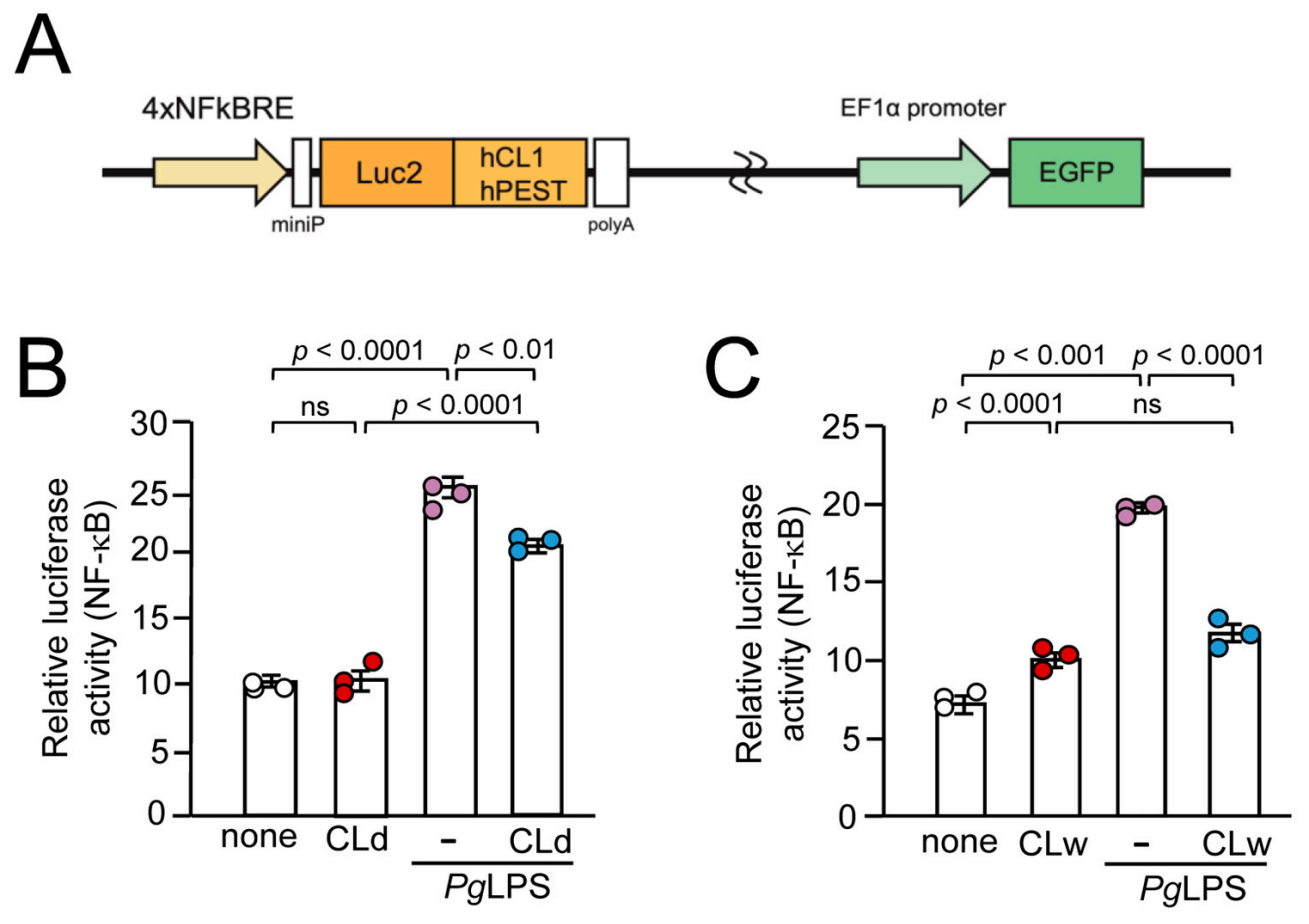
A schematic illustration of the NF-κB probe construct and effects of CL on *Pg* LPS-induced NF-κB activation in BV-2 microglia at 1 h after stimulation. (**A**) Schematic illustration of the NF-κB probe construct. (**B**) Mean relative luciferase activity of the NF-κB probe following treatment with CLd (10 μM), *Pg* LPS (30 μg/mL), or their co-application. (**C**) Mean relative luciferase activity of the NF-κB probe following treatment with CLw (100 μM), *Pg* LPS (30 μg/mL), or their co-application. “-” indicates vehicle treatment (no CL exposure, DMSO alone for CLd experiments and water for CLw experiments). Data are presented as individual data points from 3 independent experiments with the mean ± SE. Each dot represents one independent experiment. Statistical analyses were performed using one-way analysis of variance followed by Tukey’s post hoc test. ns, not statistically significant.

NF-κ transcriptional activity was quantified by measuring luciferase activity in NF-κBRE-Luc2 reporter BV-2 microglia (Figure 2A). CLd (10 μM) showed no significant effect at 1 h, while *Pg* LPS (30 μg/mL) significantly increased luciferase activity in NF-κBRE-Luc2 reporter BV-2 microglia at 1 h after stimulation (Figure 2B). Co-treatment with CLd and *Pg* LPS consistently and significantly reduced NF-κB transcriptional activity compared with *Pg* LPS alone (Figure 2B). On the other hand, CLw (100 μM) significantly increased the transcriptional activity of NF-κB at 1 h after stimulation (Figure 2C). As observed with CLd, co-treatment with CLw and *Pg* LPS consistently and significantly reduced the activation of NF-κBcompared with *Pg* LPS alone (Figure 2C).

### 2.3. Possible Inhibition of the Toll-like Receptor (TLR)2-Mediated Signaling Pathway by CL in BV-2 Microglia

**Figure 3:**
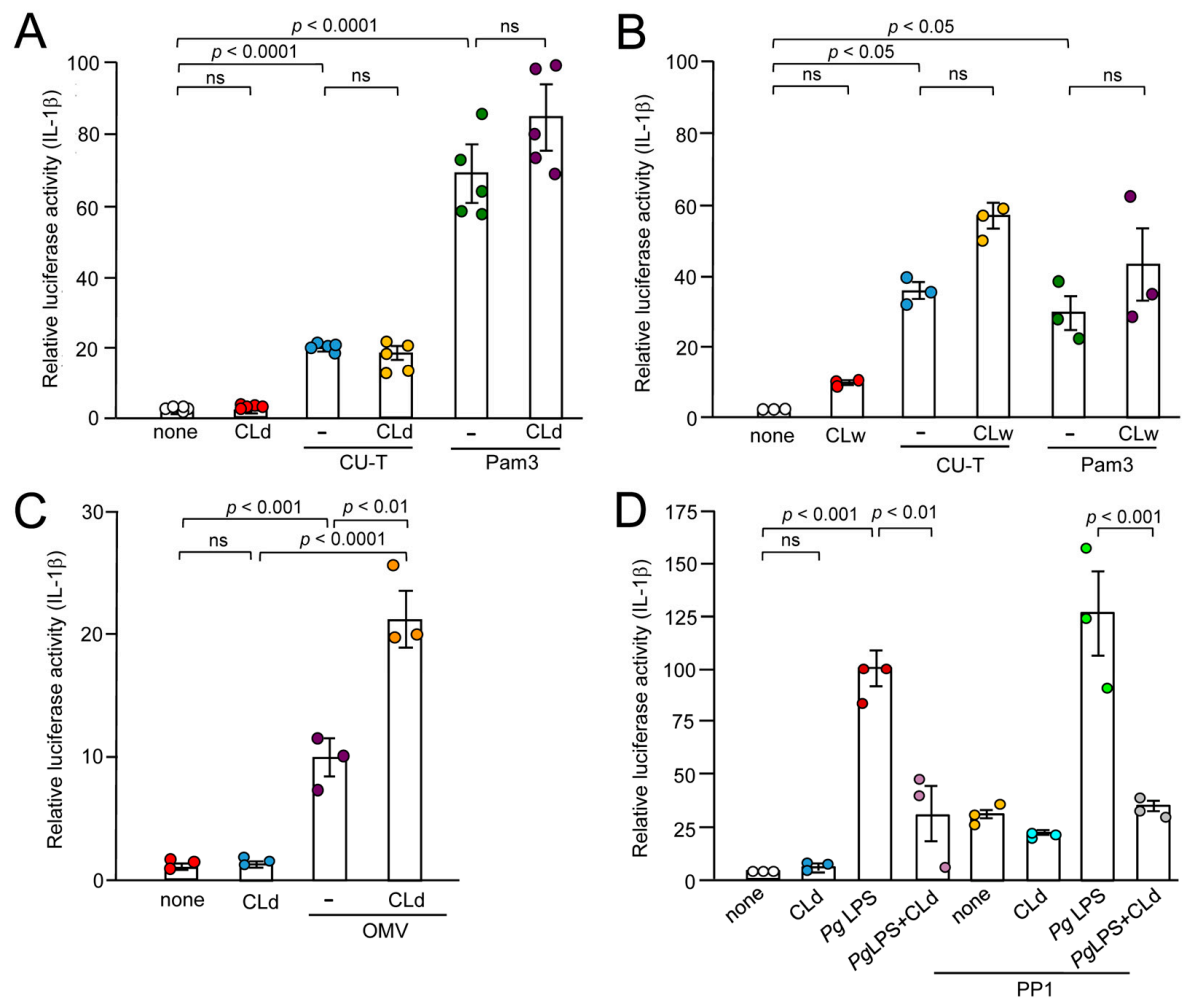
Effects of CL on IL-1β production induced by CU-T12-9, Pam_3_CSK_4_, or OMVs in BV-2 microglia at 1 h after stimulation. (**A**) Mean relative luciferase activity of the IL-1β probe following treatment with CLd (10 μM), CU-T (30 μM), Pam3 (300 nM), or their co-application. CU-T: CU-T12-9; Pam3: Pam_3_CSK_4_ (**B**) Mean relative luciferase activity of the IL-1β probe following treatment with CLw (100 μM), CU-T (30 μM), Pam3 (300 nM), or their co-application. CU-T: CU-T12-9; Pam3: Pam_3_CSK_4_ (**C**) Mean relative luciferase activity of the IL-1β probe following treatment with CLd (10 μM), OMVs (150 μg of protein/mL), or their co-application. (**D**) Mean relative luciferase activity of the IL-1β probe following treatment with CLd (10 μM), *Pg* LPS (30 μg/mL), or their co-application in the absence or presence of PP1 (30 μM). “-” indicates vehicle treatment (no CL exposure, DMSO alone for CLd experiments and water for CLw experiments). Data are presented as individual data points from 3 to 5 independent experiments with the mean ± SE. Each dot represents one independent experiment. Statistical analyses were performed using one-way analysis of variance followed by Tukey’s post hoc test. ns, not statistically significant.

One possible mechanism underlying the effect of CL on *Pg* LPS-induced IL-β production is that CL inhibits the TLR2-mediated signaling pathway. To dissect this possibility, we examined the effects of CL on TLR2 agonist-induced IL-1β production in microglia. We used CU-T12-9, a small-molecule TLR1/2 agonist, and Pam_3_CSK_4_, a lipopeptide analogue. Both CU-T12-9 (30 μM) and Pam_3_CSK_4_ (300 nM) significantly increased luciferase activity in IL-1βNluc reporter BV-2 microglia (Figure 3A,B). CLd showed no significant effect on the mean IL-1β level induced by either CU-T12-9 or Pam_3_CSK_4_ (Figure 3A). CLw also showed no significant effect on the mean IL-1β level induced by either CU-T12-9 or Pam_3_CSK_4_ (Figure 3B). Interestingly, CLd significantly increased the mean IL-1β level induced by OMVs (150 μg of protein/mL) (Figure 3C). These findings suggest that the inhibitory effect of CL on *Pg* LPS-induced IL-1β production is not mediated through inhibition of the TLR2-mediated signaling pathway in BV-2 microglia.

### 2.4. Effects of CL on CD11b-Mediated Signal Pathway in BV-2 Microglia

CD11b, a member of the integrin family, is highly expressed on monocytes, macrophages, dendritic cells and microglia. CD11b on microglia can suppress TLR activation-induced inflammatory responses by inhibiting inflammatory cytokines and increasing IL-10 by activating Src/Syk signaling [20]. CL can activate CD11b through direct binding [13]. To clarify this possibility, we examined the effects of PP1 (30 μM), a Src inhibitor, on the CLd-induced inhibition of *Pg* LPS-induced production of IL-1β in microglia. PP1 had no effect on the CLd-induced inhibition of *Pg* LPS-induced production of IL-1β in BV-2 microglia (Figure 3D).

### 2.5. Quantitation of CL Binding to Pg Lipoprotein Using an 8-Anilino-1-Naphthalenesulfonic Acid Sodium Salt (ANS-Na) Binding Assay

CL is an amphipathic peptide with a hydrophobic *N*-terminus and a hydrophilic *C*-terminus. To investigate whether CL interacts with outer membrane lipoproteins contaminating the *Pg* LPS preparation through hydrophobic interactions, ANS-Na was used to analyze the binding of hydrophobic molecules to CL. When bound to hydrophobic sites, ANS-Na can be excited to emit fluorescence, whereas unbound ANS-Na remains non-fluorescent [21]. If hydrophobic molecules occupy the hydrophobic sites of CL, ANS-Na cannot bind and consequently fluorescent intensity decreases depending on direct competition with the other bound molecule.

**Figure 4:**
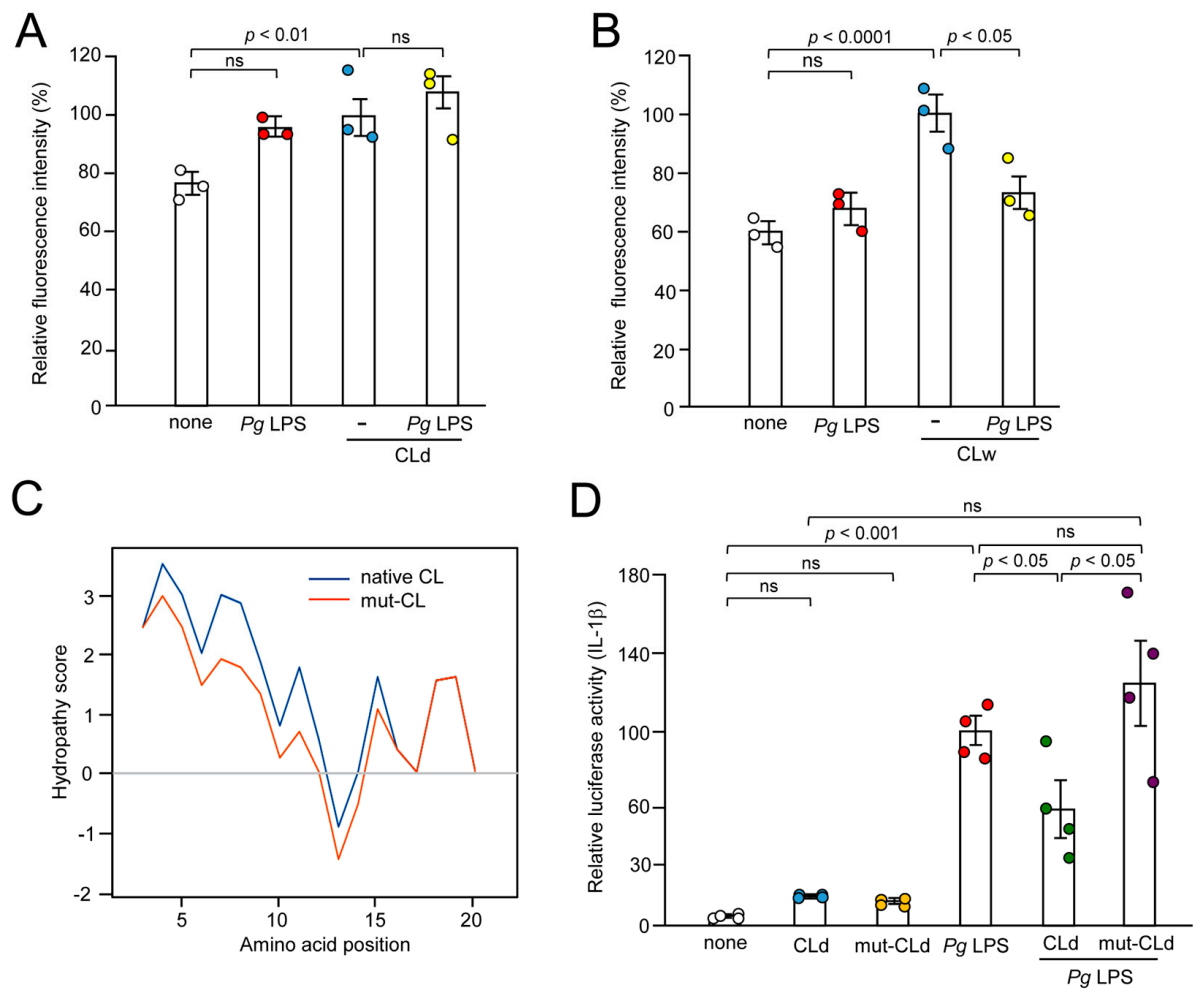
Possible hydrophobic interactions between CL and outer membrane lipoproteins contaminating the *Pg* LPS preparation. (**A**) Mean relative fluorescent intensity (%) of ANS-Na following treatment with *Pg* LPS (30 μg/mL), CLd (10 μM), or their combination. (**B**) Mean relative fluorescent intensity (%) of ANS-Na following treatment with *Pg* LPS (30 μg/mL), CLw (100 μM), or their combination. (**C**) Hydropathy plots based on the Kyte–Doolittle scale for native CL (blue line) and mutant CL (mut-CL, red line). Positive hydropathy scores indicate enrichment of hydrophobic amino acids. (**D**) Mean relative luciferase activity of the IL-1β probe following treatment with *Pg* LPS (30 μg/mL), native CLd (10 μM), mut-CLd (10 μM), or their combination. “-” indicates vehicle treatment (water alone). Data are presented as individual data points from 3 to 4 independent experiments with the mean ± SE. Each dot represents one independent experiment. Statistical analyses were performed using one-way analysis of variance followed by Tukey’s post hoc test. ns, not statistically significant.

CLd (10 μM) significantly but modestly increased the mean fluorescence intensity of ANS-Na (Figure 4A), whereas CLw (100 μM) induced a significant and marked increase (Figure 4B). *Pg* LPS (30 μg/mL) did not alter the fluorescence intensity. The mean fluorescence intensity modestly increased by CLd (10 μM) and was unaffected by co-treatment with *Pg* LPS (30 μg/mL) (Figure 4A). In contrast, the mean fluorescence markedly elevated by CLw alone was significantly decreased by approximately 26% following co-treatment with *Pg* LPS (Figure 4B), suggesting that the fluorescence of ANS-Na is markedly influenced by the solvent used. These observations suggest that CLw inhibits *Pg* LPS-induced IL-1β production by interacting with lipoproteins contaminating the *Pg* LPS preparation through hydrophobic interactions.

### 2.6. Effects of Mutant CL (Mut-CL) on Pg LPS-Induced IL-1β Production by BV-2 Microglia

To examine whether hydrophobic interactions are responsible for the inhibitory effect of CL on *Pg* LPS-induced IL-1β production, we used mut-CL in which hydrophobicity was selectively reduced by replacement of three *N*-terminal Ile residues with Ala residues. The grand average of hydropathicity (GRAVY) index value of mut-CL (0.874) was lower than that of native CL (1.106), supporting the notion that Ala substitutions reduced the overall hydrophobicity of the peptide. Furthermore, Ala substitutions reduced local hydrophobicity particularly in the *N*-terminal region of CL, as demonstrated by Kyte–Doolittle hydropathy analysis (Figure 4C). As shown in Figure 4D, mut-CLd failed to inhibit *Pg* LPS-induced IL-1β production in BV-2 microglia (Figure 4D). These results suggest that the hydrophobic *N*-terminal region of CL plays a central role in hydrophobic interactions with *Pg* outer membrane lipoproteins contaminating the *Pg* LPS preparation.

### 2.7. Three-Dimensional Structures of Native CL and Mut-CL Using AlphaFold2 Prediction

**Figure 5:**
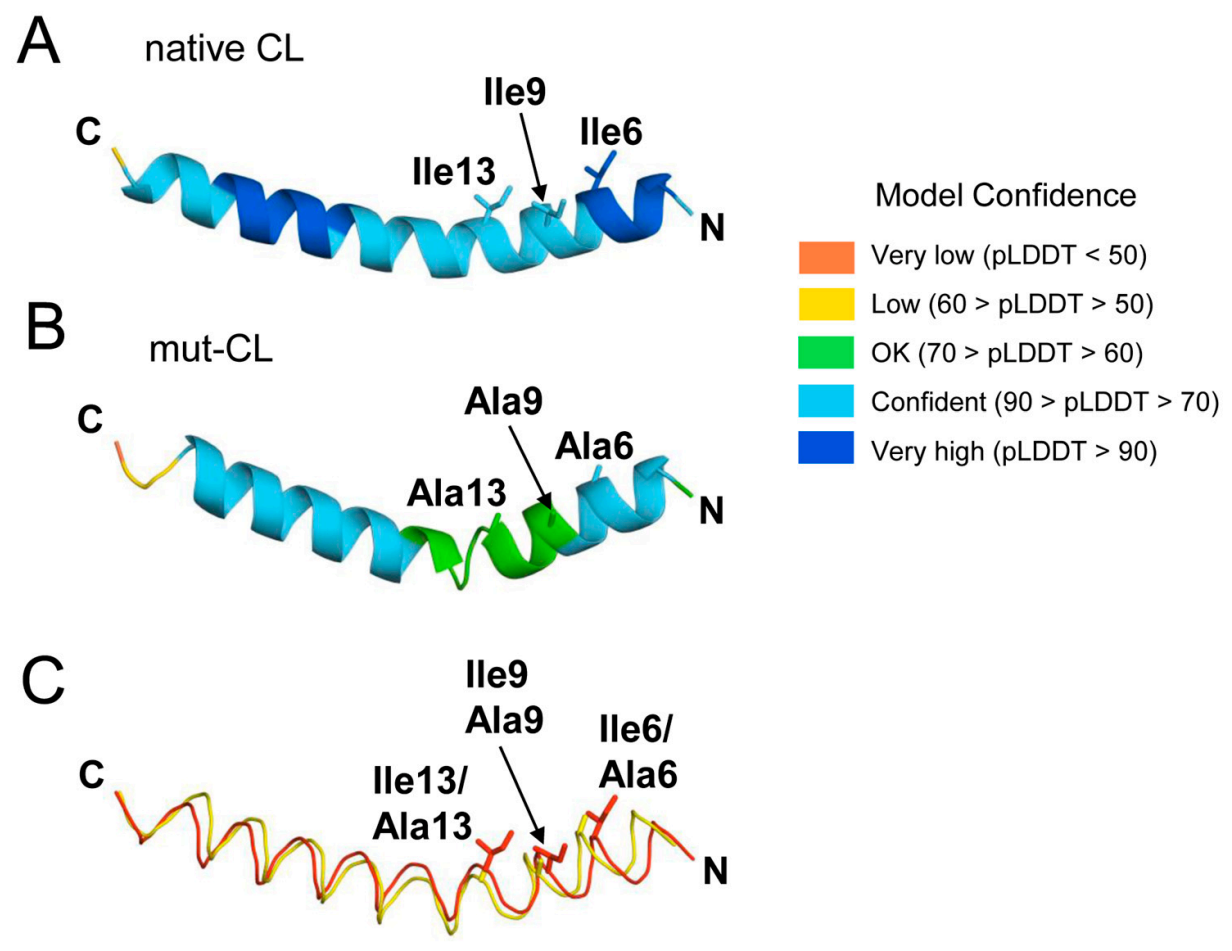
Comparison between the AlphaFold models of native CL and its mutant (mut-CL). (**A**) The AlphaFold model of CL. The structure is presented as a cartoon representation with carbon atoms colored according to pLDDT values. The side chains of Ile6, Ile9, and Ile13 are shown as stick models. (**B**) AlphaFold model of mut-CL. The structure is presented as a cartoon representation with carbon atoms colored according to pLDDT values. The side chains of Ala6, Ala9, and Ala13 are shown as stick models. Regions with pLDDT > 90 are colored blue, regions with 70 < pLDDT < 90 are colored cyan, regions with 60 < pLDDT < 70 are colored green, regions with 50 < pLDDT < 60 are colored yellow, and regions with pLDDT < 50 are colored red. (**C**) Structural comparison between native CL and mut-CL. The Cα atoms in the native CL model (red) were superimposed onto those of mut-CL (yellow) using maximal structure alignment. The figure was generated using PyMOL 3.1 [22].

Finally, to elucidate whether mut-CL maintains overall structural properties, we compared the tertiary structures of native CL and mut-CL using AlphaFold prediction. The AlphaFold model of native CL was generated with high confidence, with an average predicted Local Distance Difference Test (pLDDT) score of 85.48 across all Cα atoms (Figure 5A). In the model, Ile2–Asn34 forms one α helix. To investigate potential structural changes resulting from replacing Ile6, Ile9, and Ile13 with Ala, the mut-CL model was generated, with an average pLDDT score for all Cα atoms of 70.45 (Figure 5B). In the mutant, two α helices (α1 and α2) were identified. Ile2–Ala13 forms the α1 helix with an average pLDDT score of 70.79, while Val16–Lys32 forms the α2 helix with an average pLDDT score of 81.47. Although the region from Ala9 in the α1 helix to Ile17 in the α2 helix shows relatively low confidence (an average pLDDT score of 64.75), the predicted mutant structure appeared sufficiently reliable for further structural interpretation. The native CL can be superimposed on the mut-CL with a root mean square deviation of 1.32 Å for 35 *C*α atoms, suggesting that the overall structures of native CL and mut-CL are largely conserved (Figure 5C).

## 3. Discussion

In this study, we found that CL significantly inhibited IL-1β production by BV-2 microglia following stimulation with *Pg* LPS. *Pg* LPS obtained using the classical hot phenol extraction method contains outer membrane lipoproteins. These contaminating *Pg* outer membrane lipoproteins are thought to be primarily responsible for IL-1β production, because lipase-treated *Pg* LPS induced only a small amount of IL-1β in BV-2 microglia. Furthermore, *Pg* LPS-induced IL-1β production was completely suppressed by C29, a TLR2 inhibitor, but not by TAK-294, a TLR4 inhibitor [11,23]. These findings suggest that CL significantly inhibited IL-1β production induced by *Pg* outer membrane lipoproteins in BV-2 microglia.

Both CLd and CLw significantly suppressed *Pg* LPS-induced IL-1β production by 35–60% and NF-κB activation by 20–40%. It was noted that CLd alone had no significant effect on NF-κB transcriptional activity, whereas CLw alone significantly increased NF-κB activity. In the present study, CLw was used at 100 μM, whereas CLd was used at 10 μM, based on their relative bioactivities [19]. Therefore, the difference in NF-κB transcriptional activity observed between CLw and CLd is likely attributable to this difference in effective CL concentration and bioavailability. Thus, the apparent increase in NF-κB activity induced by CLw may reflect the higher nominal concentration used, whereas CLd at 10 μM did not significantly affect NF-κB activity under these experimental conditions. Importantly, the lack of NF-κB activation by CLd does not necessarily indicate an inhibitory effect of DMSO on CL activity.

There are at least three possible mechanisms underlying the inhibitory effects of CL on *Pg* LPS-induced IL-1β production in BV-2 microglia: (1) CL inhibits the TLR2 signaling pathway, (2) CL inhibits *Pg* LPS-induced IL-1β production by binding to CD11b, and (3) CL directly interacts with *Pg* outer membrane lipoproteins contaminating the *Pg* LPS preparation through hydrophobic interactions. The present study does not support the first possibility, because neither CLd nor CLw showed significant effects on IL-1β production induced by the TLR2 agonists such as CU-T12-9 and Pam_3_CSK_4_ in BV-2 microglia. The second possibility also appears unlikely, because PP1 failed to block the inhibitory effect of CL on *Pg* LPS-induced IL-1β production. Although CLd significantly enhanced OMV-induced IL-1β production, the underlying mechanism remains unclear. Because OMVs contain multiple bacterial components, including lipoproteins and other microbial molecules, their interaction with CL may differ from that with purified TLR2 agonists. In fact, OMVs induced IL-1β production in BV-2 microglia via a TLR2-independent pathway [11,12]. In addition, whether the enhancing effect of CLd on OMV-induced IL-1β production is also observed with water-solubilized CL remains unclear and requires further investigation.

There is accumulating evidence that CL can directly bind host proteins, including the integral CD11b expressed on microglia, thereby promoting protective neuroinflammatory responses during cerebral *C. albicans* infection [13]. Albumin and heparin have also been identified as host proteins capable of neutralizing CL activity through hydrophobic interactions [24,25]. These interactions reduce the toxic activity of CL. Therefore, direct hydrophobic interactions between CL and *Pg* outer membrane lipoproteins contaminating the *Pg* LPS preparation may represent the most plausible mechanism underlying the inhibitory effect of CL on *Pg* LPS-induced IL-1β production.

ANS-Na exhibits weak fluorescence in a highly polar environment such as water, whereas its fluorescence markedly increases in hydrophobic environments. This is because non-radiative decay of ANS-Na is favored in polar solvents but suppressed in hydrophobic environments, resulting in enhanced fluorescence intensity [21]. In the present study, CLw markedly increased the fluorescence intensity of ANS-Na, whereas CLd induced only a modest increase. In addition, lipoproteins contaminating the *Pg* LPS preparation alone did not affect ANS-Na fluorescence. CLw forms insoluble microparticles, while CLd is fully soluble [19]. Since ANS-Na preferentially binds to hydrophobic pockets, these findings suggest that CLw forms higher-order hydrophobic structures that are absent or less developed in either CLd or lipoproteins contaminating the *Pg* LPS preparation [19,26]. Furthermore, lipoproteins contaminating the *Pg* LPS preparation are likely to interact with these hydrophobic structures generated by CLw aggregation, thereby significantly attenuating the CLw-induced ANS-Na fluorescent intensity. To further evaluate the role of hydrophobic interactions, we used mut-CL, in which three *N*-terminal Ile residues were substituted with Ala residues to selectively reduce hydrophobicity while preserving the overall peptide structure. As expected, mut-CLd failed to inhibit *Pg* LPS-induced IL-1β production in BV-2 microglia, further supporting the idea that hydrophobic interactions mediated by the *N*-terminal region are critical for the inhibitory effects of CL.

Bacterial lipoproteins are well-characterized ligands for TLR2-TLR1 heterodimers because of their well-defined chemical structures. The cysteine residue of Gram-negative bacterial lipoproteins is triacylated at the *N*-terminus. TLR1 possesses a hydrophobic channel that accommodates the third acyl chain, thereby enabling TLR2-TLR1 heterodimers to recognize triacylated lipoproteins [27]. The *Pg* strain ATCC 33277 expresses several outer membrane triacylated lipoproteins, including PG1828, PG1881, and RagB. Both PG1828 and RagB have been reported to activate TLR2 [28,29,30]. PG1881 is an OMV-specific lipoprotein and promotes *Pg* aggregation on red blood cells [31], although its ability to activate TLR2 remains unclear. *Pg* LPS prepared from PG1828-deficient mutants exhibits attenuated TLR2 activation, and lipoprotein lipase treatment reduces TLR2/TLR1 activation by *Pg* LPS [28]. Taken together, these findings suggest that PG1828 may be a major contributor to TLR2-dependent IL-1β production in BV-2 microglia. Therefore, CL may attenuate *Pg* lipoprotein-induced IL-1β production through hydrophobic interactions with the lipid moieties of PG1828. Further studies are required to elucidate the direct interaction between CL and PG1828.

Although the present study demonstrates hydrophobic interactions between CL and *Pg* lipoproteins under in vitro conditions, the extent to which this mechanism occurs in vivo remains to be determined. The brain extracellular environment contains host hydrophobic molecules, including albumin and lipoproteins, that could potentially compete with bacterial lipoproteins for CL binding. However, because CL is released locally during *Candida* infection, direct interactions between microbial virulence factors may occur before extensive association with host proteins. Moreover, albumin concentrations in the healthy brain parenchyma are relatively low because of the blood–brain barrier, although they may increase under pathological conditions. Therefore, future studies using primary microglia, cerebrospinal fluid-containing culture systems, and animal models will be necessary to determine whether host proteins influence CL–*Pg* lipoprotein interactions under physiological conditions and to establish the clinical relevance of this mechanism. Nevertheless, our findings identify a previously unrecognized mechanism by which direct interactions between microbial virulence factors may modulate innate immune signaling, providing a conceptual framework for understanding polymicrobial infections in neuroinflammatory diseases.

The present study has several limitations. First, although validated luciferase-based reporter assays were used to evaluate IL-1β production and NF-κB activation, further studies should confirm these findings using conventional protein-based methods, such as ELISA and Western blotting. Second, all experimental findings were obtained from *in vitro* experiments using BV-2 microglia, an immortalized microglial cell line. Therefore, further studies using primary microglia and *in vivo* models, including intracerebral co-infection models with *Pg* and *C. albicans,* are needed to validate the physiological relevance of the present findings.

## 4. Materials and Methods

### 4.1. Reagents

Synthetic native CL (SIIGIIMGILGNIPQVIPQVIQIIMSIVKAFKGNK) was purchased from Peptide Institute Inc. (Osaka, Japan). mut-CL (I6A, I9A, I13A), in which three *N*-terminal Ile residues were substituted with Ala residues (SIIGIAMGALGNAPQVIPQVIQIIMSIVKAFKGNK), was synthesized by GL Biochem Ltd. (Shanghai, China). *Pg* LPS (cat: tlrl-pglps) and lipase-treated *Pg* LPS (cat: tlrl-ppglps), which was prepared by enzymatic removal of lipoproteins, were purchased from InvivoGen (San Diego, CA, USA). PP1, a Src inhibitor, was purchased from Cayman Chemical (Ann Arbor, MI, USA). CU-T12-9, a TLR2/TLR1 agonist, was purchased from Selleck Biotechnology (Yokohama, Japan). Pam_3_CSK_4_ was purchased from R&D Systems (Minneapolis, MN, USA). ANS-Na was purchased from Tokyo Chemicals Industry Co., Ltd. (Tokyo, Japan).

### 4.2. Cell Culture

BV-2 cells, a murine microglial cell line [32] widely used as an alternative to primary microglia [33,34], were used in this study. BV-2 microglia were cultured in Dulbecco’s modified Eagle’s medium (Thermo Fisher Scientific, Waltham, MA, USA) supplemented with 5% fetal bovine serum, penicillin, and streptomycin.

### 4.3. Establishment of Reporter BV-2 Microglia

To establish the IL-1βNluc reporter BV-2 microglia, we infected the cells with a lentiviral vector carrying the Nluc probe and selected EGFP-positive cells, as described previously [35]. The luciferase activity was only induced when proteolytic pro-IL-1β processing occurred because the Nluc luciferase was fused with the sequence of IL-1β (*il1b* 17-216), which undergoes proteolytic cleavage by proteases including caspase-1. Furthermore, the C-terminus of *il1b* 17-216 was fused with two protein destabilization sequences (hCL1 and hPEST) (Figure 1A), thus allowing stabilization of the Nluc luciferase protein only after proteolytic processing of IL-1β.

To establish NF-κBRE-Luc2 reporter BV-2 microglia, the *NF-κBRE-Luc2* construct consisted of four tandem repeats of the NF-κB response element sequence, followed by a mini P promoter driving the Luc2 gene fused with CL1 and PEST sequences, along with an SV40 poly(A) signal. Two copies of the human 5′ β-globin insulator sequence are positioned upstream and downstream of the expression cassette. The construct was incorporated into a lentiviral transfer plasmid (*CSII-NF-κBRE-Luc2*), which was co-transfected into HEK293T cells together with the packaging plasmids *pCAG-HIVgp*, *pCMV-VSV-G*, and *pRSV-Rev* (Figure 2A). The culture supernatant was collected 48 h after transfection and filtered through a 0.45 μm pore size filter. Viral particles were concentrated 100-fold by polyethylene glycol precipitation. BV-2 microglia were infected with the viral vector, and a single-cell clone showing a robust response to LPS stimulation was selected and used for subsequent experiments.

### 4.4. Bacterial Culture and Isolation of OMVs

*Pg* ATCC 33277 was maintained as previously described [8]. Detailed methods for preparing OMVs were previously reported [8]. Briefly, *Pg* culture media were centrifuged to remove bacterial cells. The supernatant was filtered through a Millipore filter (pore size 0.22 μm, Millipore, Burlington, MA, USA) and ultracentrifuged. The pellet containing OMVs was collected and resuspended in phosphate-buffered saline. The protein concentration of OMVs was measured using a Pierce^TM^ BCA protein assay kit (Thermo Fisher Scientific Inc., Waltham, MA, USA) and added to BV-2 microglia at a final concentration of 150 μg of protein/mL.

### 4.5. Measurement of the Luciferase Activity

IL-1βNluc and NF-κBRE-Luc2 reporter BV-2 microglia were plated in 96-well white culture plates at a density of 5 × 10^4^ cells per well. After overnight culture, drug treatments were performed, and luciferase activities of IL-1βNluc and NF-κBRE-Luc2 reporter BV-2 microglia were measured using a luminometer (GloMax; Promega Corp., Madison, WS, USA) with a Nano-Glo^®^ luciferase assay system (N1110; Promega Corp.) and a One-Glo^TM^ Luciferase assay system (E6110; Promega Corp.), respectively, according to the manufacturer’s instructions. Luciferase activity was measured as relative light units (RLU) in cells and culture media. Each condition was repeated in triplicate on the same plate, and at least three independent experiments were performed.

### 4.6. Assay for Cell Survival

Cell viability was assessed using Cell Counting Kit-8 (Dojindo, Kumamoto, Japan), based on WST-8 reduction by mitochondrial dehydrogenases, according to the manufacturer’s instructions, and 0.1% Triton X-100 was used as a positive control. Absorbance was measured at a wavelength of 450 nm with a microplate reader.

### 4.7. ANS-Na Binding Assay for Quantification of CL Binding

Assay for CL binding to hydrophobic pockets of lipoproteins contaminating the *Pg* LPS preparation was based on the ANS-Na competition assay described previously [21]. First, 30 μg/mL *Pg* LPS was preincubated in 0.1 M glycylglycine buffer (pH 7.4) with either 10 μM CLd or 100 μM CLw at 37 °C. After 1 h, 10 μM of ANS-Na was added, and samples were incubated for 20 min at room temperature. The fluorescence intensity of ANS-Na was measured in a plate reader at an excitation wavelength of 373 nm, and the fluorescence emission was recorded at 474 nm.

### 4.8. GRAVY and Hydropathy Scores

GRAVY and hydropathy scores were calculated using Expasy ProtParam (https://web.expasy.org/protparam/, accessed on 20 May 2026) and TCDB Hydropathy Plot (https://www.tcdb.org/progs/hydropathy.php, accessed on 20 May 2026), respectively.

### 4.9. AlphaFold Predictions

Structures of CL were predicted using AlphaFold2 [36] implemented in ColabFold with default settings and 48 recycles. For analysis of the wild-type candidalysin, the amino acid sequence, SIIGIIMGILGNIPQVIPQVIQIIMSIVKAFKGNK, was loaded on the Colab server (https://colab.research.google.com/github/sokrypton/ColabFold/blob/main/AlphaFold2.ipynb, accessed on 7 March 2026). For analysis of the mutant, the sequence, SIIGIAMGALGNAPQVIPQVIQIIMSIVKAFKGNK, was used to predict its structure. Five models were generated for each protein, and the best model was selected for further analysis.

### 4.10. Statistical Analyses

Data are presented as the mean ± SE. Statistical analyses were performed using one-way analysis of variance followed by Tukey’s post hoc test with GraphPad Prism8 (GraphPad Software, Inc., San Diego, CA, USA) software package. *p* < 0.05 was considered statistically significant.

## Data Availability

The original contributions presented in this study are included in the article. Further inquiries can be directed to the corresponding author.

## Abbreviations

The following abbreviations are used in this manuscript: AD: Alzheimer’s disease; ANS-Na: 8-anilino-1-naphthalenesulfonic acid sodium salt; BBB: blood–brain barrier; CL: candidalysin; CLd: CL dissolved in DMSO; CLw: CL dissolved in water; DMSO: dimethyl sulfoxide; GRAVY: the grand average of hydropathy; IL-1b: interleukin-1b; LPS: lipopolysaccharide; NF-kB: nuclear factor-kB; OMVs: outer membrane vesicles; *Pg*: *Porphyromonas gingivalis*; RLU: relative light units; SE: standard error; TLR: Toll-like receptor.
